# Transcriptomic Remodeling of Light Harvesting and Photosystem Genes in *Acaryochloris marina* Under a Low-Irradiance Far-Red Versus High-Irradiance White Light

**DOI:** 10.3390/plants15111605

**Published:** 2026-05-23

**Authors:** Abraham Peele Karlapudi, Vuyyuru Kesavi Himabindhu, Divya Kaur

**Affiliations:** 1 Department of Biotechnology, Vignan’s Foundation for Science, Technology and Research, Guntur 522213, Andhra Pradesh, India; drapk_bt@vignan.ac.in (A.P.K.);; 2 Department of Chemistry, Brock University, St. Catharines, ON L2S 3A1, Canada

**Keywords:** *Acaryochloris marina*, chlorophyll d, low-irradiance far-red light, phycobiliproteins, chlorophyll-binding antenna, transcriptomics, RNA-seq, chromatic acclimation type 5 (CA5), cyanobacteria, co-expression network analysis

## Abstract

*Acaryochloris marina* is a distinctive cyanobacterium that uses chlorophyll *d* as its primary photosynthetic pigment and possesses two major light-harvesting systems: membrane-integral chlorophyll-binding Pcb/CBP complexes and water-soluble phycobiliproteins. How these antenna systems respond at the transcriptome level to contrasting light environments remains incompletely characterized. Here, we re-analyzed a publicly available RNA-seq dataset for *A. marina* MBIC11017 (NCBI BioProject PRJNA1130970), comparing cells grown under low-irradiance far-red light (LL-FR; 1.5–2 µmol photons m^−2^ s^−1^, 710-nm peak) and high-irradiance white light (HL-WL; 30–35 µmol photons m^−2^ s^−1^). Because light quality and irradiance both differ in this experimental design, the two effects cannot be separated; all transcriptional changes are therefore interpreted as responses to the combined LL-FR versus HL-WL contrast rather than to far-red wavelength alone. Of 8439 expressed genes, 1810 (21.4%) were significantly differentially expressed (adjusted *p* < 0.05). Using GFF-verified locus tags which corrected mis-annotations propagated in earlier analyses, the PS-I core gene set showed a mean log_2_ fold-change of +1.96 (3.9-fold; 11/11 loci significant), whereas the PS-II core gene set showed a mean log_2_ fold-change of +1.10 (2.1-fold; 12/20 loci significant). Light-harvesting genes showed the strongest response: 17/18 phycobiliprotein-pathway genes in KEGG amr00196 were upregulated, together with multiple putative Pcb/CBP loci (mean antenna log_2_FC = +3.51; 11.4-fold). Weighted gene co-expression network analysis placed the antenna-associate genes examined here within a module positively correlated with the LL-FR condition (r = 0.802, *p* = 0.017), and STRING analysis supported an enriched network of predicted or known protein associations (1115 nodes, 4763 edges; PPI enrichment *p* < 1.0 × 10^−16^). Recent matched-irradiance experiments indicate that, at equal photon flux, far-red wavelengths reduce phycobilisome content relative to white light. The transcriptional pattern reported here is therefore most parsimoniously interpreted as predominantly a low-irradiance response, with possible wavelength-associated CA5 contributions that cannot be isolated in the present design. Overall, the analysis reveals coordinated transcript-level changes across plasmid-encoded reacquired phycobiliprotein genes, chromosomal Pcb/CBP loci, chlorophyll biosynthesis genes, and photosystem core genes, consistent with coordinated regulation of light-harvesting components in *A. marina*.

## 1. Introduction

Photosynthetic organisms use light-harvesting antenna systems to absorb light energy and transfer excitation to the reaction centers of photosystems I and II. The absorption properties of these antenna systems help determine which light environments can support growth. Most oxygenic phototrophs rely on chlorophyll *a* and associated pigments that absorb mainly in the visible region of the spectrum. However, some cyanobacteria have evolved strategies that extend oxygenic photosynthesis into the far-red region, allowing them to grow in habitats where visible light is limited but longer wavelengths remain available [1,2,3].

*Acaryochloris marina* is one of the most remarkable examples of this adaptation. Unlike most cyanobacteria, which use chlorophyll *a* as their primary photosynthetic pigment, *A. marina* uses chlorophyll *d* (Chl *d*) as its dominant pigment, enabling efficient absorption of far-red light above 700 nm [4,5]. This spectral specialization allows the organism to occupy ecological niches such as shaded microbial communities and environments beneath other phototrophs, where shorter wavelengths have already been absorbed [6,7]. Genomic studies have further shown that *A. marina* has an expanded set of photosynthesis-related genes, including duplicated genes associated with light-harvesting and photosynthetic function, which may support its ability to adapt to different light conditions [8,9,10].

A distinctive feature of *A. marina* is its possession of two different light-harvesting antenna systems. One consists of membrane-integral Pcb (prochlorophyte chlorophyll-binding) proteins, also referred to as chlorophyll-binding proteins (CBPs). These proteins are related to the CP43 antenna subunit of photosystem II [11,12,13] and function as membrane-associated chlorophyll-binding complexes that expand the absorption cross-section of the photosystems [14,15]. In addition, some strains of *A. marina* contain genes encoding water-soluble phycobiliproteins. Comparative genomic studies suggest that these genes were reacquired by horizontal gene transfer, indicating a secondary acquisition of phycobiliprotein-based light harvesting within this lineage [16,17]. Notably, the strain MBIC11017 retains genes encoding C-phycocyanin, whereas other *Acaryochloris* strains lack these phycobiliprotein components [17,18]. The coexistence of chlorophyll-binding Pcb proteins and phycobiliproteins therefore gives *A. marina* an unusual hybrid antenna system among oxygenic phototrophs.

This dual antenna architecture is linked to a specialized form of chromatic acclimation (CA5), in which the composition and expression of light-harvesting proteins change in response to light quality [16,18,19]. Recent studies of *A. marina* MBIC11017 grown under contrasting light conditions suggest that horizontally acquired phycobiliprotein genes have become integrated into pre-existing regulatory networks involved in light harvesting [18,20]. However, the extent to which phycobiliprotein genes and chlorophyll-binding antenna genes respond in a coordinated manner at the transcriptome level remains unclear.

In this study, we re-analyzed a publicly available RNA-seq dataset for *A. marina* MBIC11017 (NCBI BioProject PRJNA1130970) to examine genome-wide transcriptional responses under low-irradiance far-red light and high-irradiance white light. We focused on whether genes encoding phycobiliproteins and chlorophyll-binding antenna proteins show coordinated expression under these contrasting conditions. By combining differential expression, pathway enrichment, and network-based analyses, we evaluated whether antenna-related transcriptional remodeling is a central feature of the *combined* LL-FR versus HL-WL response in *A. marina*.

## 2. Results

### 2.1. Read Mapping and Library Statistics

The eight RNA-seq libraries generated a total of 190,638,828 fragment pairs, of which 78,070,934 reads (41.0%) were assigned to annotated gene features following alignment to the *Acaryochloris marina* MBIC11017 reference genome (Table 1; Supplementary Figure S1). Sequencing depth and mapping efficiency were broadly comparable across samples, with assignment rates ranging from 34.0% to 48.7%. Normalization using DESeq2 yielded size factors ranging from 0.55 to 1.38, indicating moderate variation in library size but no systematic bias across experimental conditions.

### 2.2. Global Transcriptomic Patterns

**Figure 1:**
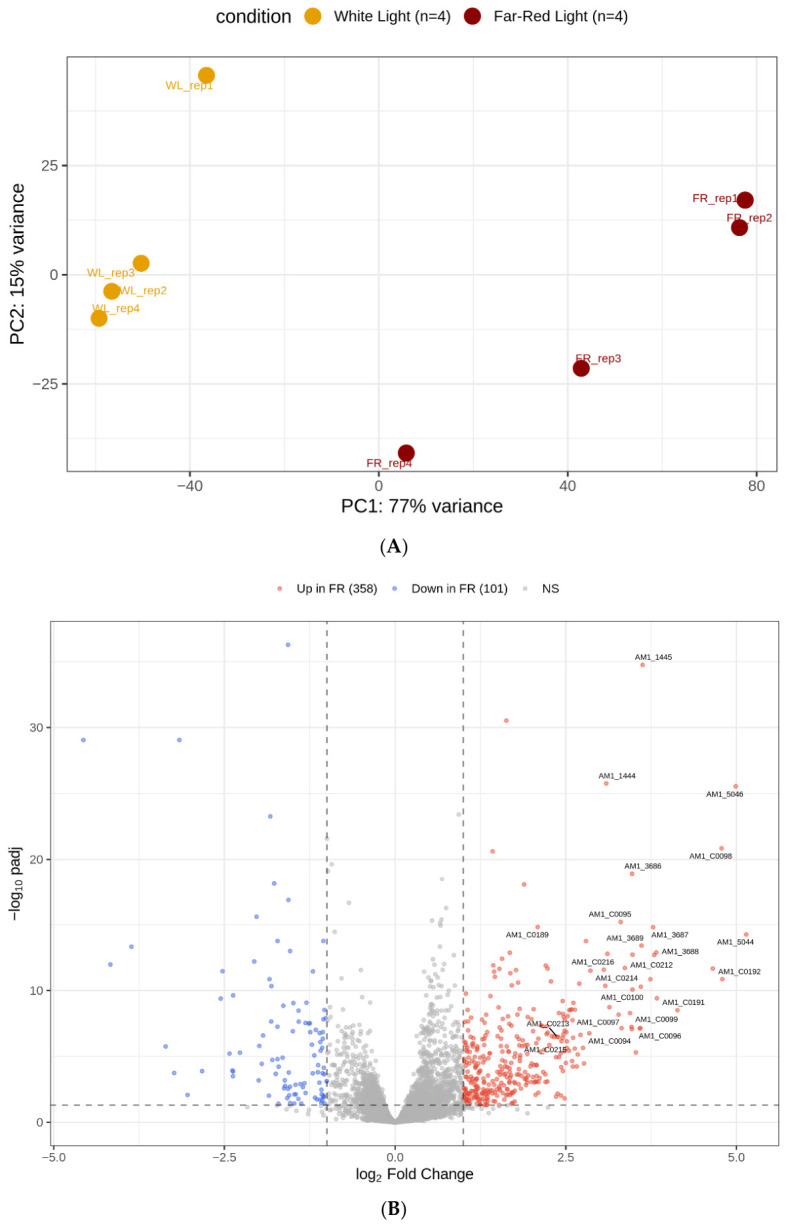
(**A**) Principal component analysis (PCA) of RNA-seq libraries from *Acaryochloris marina* MBIC11017 grown under HL-WL and LL-FR conditions. (**B**) Volcano plot of differential gene expression between LL-FR and HL-WL conditions in *Acaryochloris marina* MBIC11017. In panel (**B**), the dashed horizontal line indicates an adjusted *p*-value threshold of 0.05 (−log_10_ padj = 1.30), and the dashed vertical lines indicate log_2_ fold-change thresholds of ±1.

Principal component analysis (PCA) of variance-stabilized expression values showed a clear separation between samples grown under high irradiance white light (HL-WL) and low irradiance far-red light (LL-FR) conditions (Figure 1A; Supplementary Figure S1). The first principal component (PC1), which explained 78.0% of the total variance, separated the two light environments, whereas the second principal component (PC2), which explained 15.0% of the variance, reflected the variability among biological replicates. Replicates from the LL-FR condition were slightly more dispersed along PC2, indicating that there was more transcriptional variation within the LL-FR condition.

### 2.3. Differential Expression Overview

Differential expression analysis identified extensive transcriptional differences between the two conditions. Of the 8439 genes tested, 1810 genes (21.4%) were significantly differentially expressed at an adjusted *p* value below 0.05. Among these genes, 1135 were upregulated and 675 were downregulated under LL-FR relative to HL-WL. When a stricter threshold of |log_2_FC| > 1 was applied, 358 genes showed strong upregulation and 101 genes showed strong downregulation. The overall distribution of fold changes and statistical significance is illustrated in the volcano plot (Figure 1B; Supplementary Figures S2–S4), where several of the most strongly induced genes correspond to members of light-harvesting antenna families.

### 2.4. Coordinated Upregulation of the Photosynthetic Apparatus in the LL-FR Versus HL-WL Contrast

**Figure 2:**
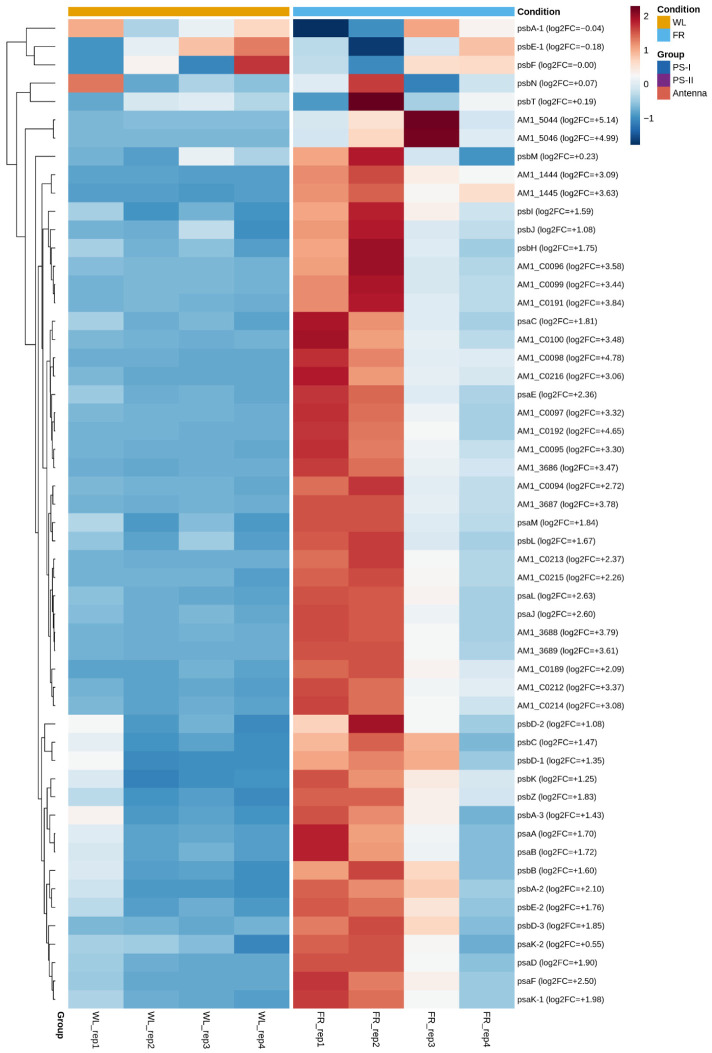
Heatmap of normalized expression values for selected photosystem I, photosystem II, and light-harvesting genes in *Acaryochloris marina* grown under HL-WL and LL-FR conditions. Columns represent individual RNA-seq libraries, and rows represent genes. Values were normalized with DESeq2 and scaled by gene.

Despite widespread transcriptional changes across the genome, genes encoding the core subunits of photosystem I and photosystem II were significantly upregulated under LL-FR relative to HL-WL. The PS-I gene set (11 gene loci, encoding 10 subunit types) showed a mean log_2_ fold change of +1.963 (3.9-fold; all 11/11 loci significant, *p* < 0.001), while the PS-II gene set (20 gene loci, encoding 15 subunit types) showed a mean log_2_ fold change of +1.103 (2.1-fold; 12/20 loci significant, *p* < 0.001). This pattern is also evident in the heatmap of selected photosynthesis-related genes (Figure 2), where both core photosystem genes and several antenna-associated genes are upregulated under LL-FR conditions, with antenna genes showing the strongest response.

### 2.5. Upregulation of Phycobiliprotein Genes

The strongest transcriptional response in the LL-FR versus HL-WL contrast occurred within the KEGG photosynthesis–antenna protein pathway (amr00196). This pathway contains 18 phycobiliprotein-related genes, of which 17 were significantly upregulated under LL-FR conditions (Table 2). These genes encode multiple components of the phycobilisome, including C-phycocyanin α and β subunits (cpcA and cpcB), rod–core linker proteins (cpcC), rod cap linkers (cpcD), rod–rod linkers (cpcG), and proteins involved in chromophore attachment (cpcE and cpcF). Expression increases ranged from approximately 2-fold to 27.5-fold. Nearly all of these genes are located on the plasmid pREB3, consistent with previous genomic studies indicating that phycobiliprotein genes in *A. marina* were reacquired through horizontal gene transfer. The only chromosomal gene within this pathway, apcB (AM1_2376), which encodes an allophycocyanin β subunit, was also significantly upregulated (2.5-fold). Gene-set analysis confirmed strong coordinated induction of the pathway (Wilcoxon *p* = 8.33 × 10^−17^; permutation *p* < 10^−5^).

### 2.6. Additional Light-Harvesting and Pigment Biosynthesis Genes

**Figure 3:**
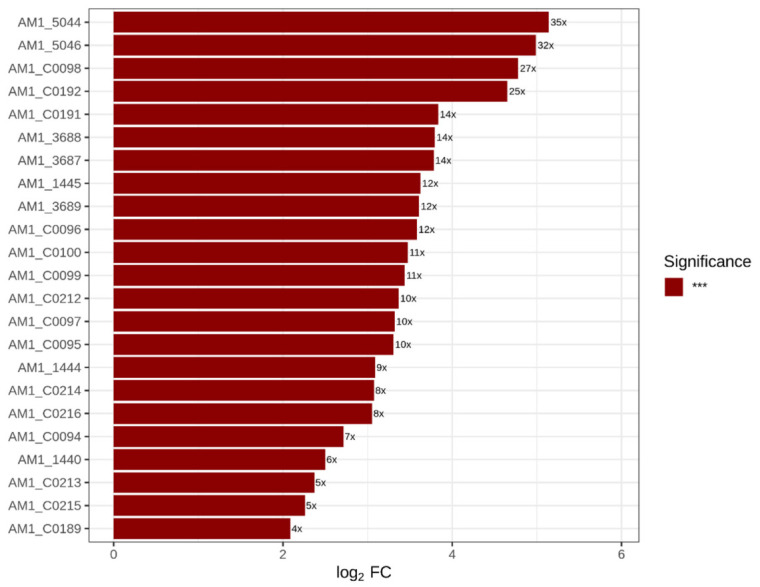
Log2 fold changes in significantly upregulated genes associated with light harvesting and pigment biosynthesis in *Acaryochloris marina* under LL-FR relative to HL-WL conditions. Genes shown include members of the KEGG photosynthesis–antenna protein pathway together with additional light-harvesting and pigment-associated genes listed in Table 2. *** indicates statistical significance at adjusted *p* < 0.001 (Benjamini–Hochberg method).

In addition to the KEGG-defined phycobiliprotein pathway, several genes associated with light harvesting and pigment biosynthesis were strongly induced under LL-FR conditions (Table 2; Figure 3). Five chromosomally encoded loci (AM1_3686–3689 and AM1_5044) showed particularly strong induction, with expression increases ranging from approximately 11-fold to 35-fold. Although annotated as hypothetical proteins in RefSeq, their genomic context and expression patterns are consistent with chlorophyll-binding Pcb/CBP antenna proteins described previously in *A. marina*. Genes involved in chlorophyll biosynthesis were also significantly upregulated. The chlL and chlN genes, which encode subunits of the dark-operative protochlorophyllide oxidoreductase involved in chlorophyll synthesis, showed expression increases of 12.3-fold and 8.5-fold, respectively. In addition, the PS-II extrinsic protein psbU (AM1_5046), which contributes to stabilization of the oxygen-evolving complex, showed strong induction (31.8-fold). Several additional plasmid-encoded genes of unknown function (AM1_C0095, AM1_C0097, AM1_C0189, and AM1_C0214) were also upregulated and exhibited expression patterns similar to those of the phycobiliprotein genes.

### 2.7. KEGG Pathway Enrichment Analysis

**Figure 4:**
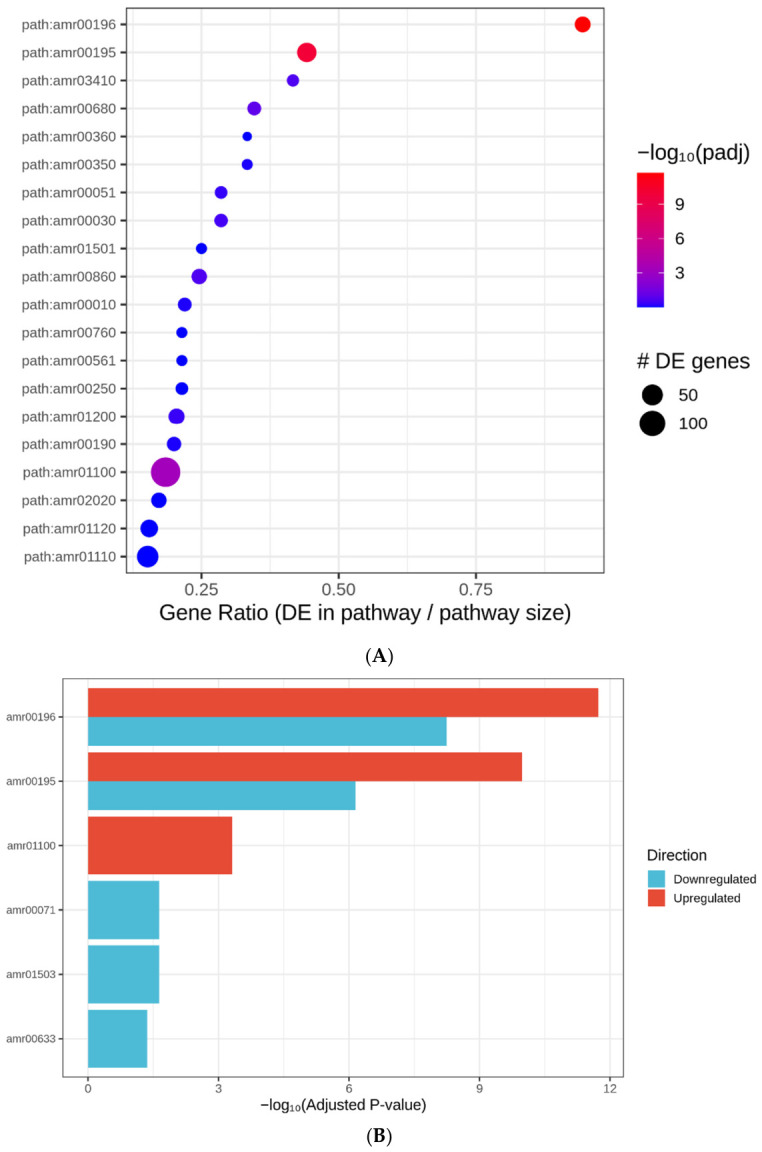
(**A**) KEGG pathways significantly enriched among genes upregulated under LL-FR relative to HL-WL in *Acaryochloris marina*. Bubble size indicates the number of differentially expressed genes in each pathway, and color indicates significance as −log10(adjusted *p* value). (**B**) Summary of significantly enriched KEGG pathways among differentially expressed genes in *Acaryochloris marina* under LL-FR relative to HL-WL conditions.

KEGG pathway enrichment analysis further highlighted the importance of light-harvesting processes in the LL-FR versus HL-WL transcriptional response. Three pathways were significantly enriched among upregulated genes (adjusted *p* < 0.05; Table 3; Supplementary Figures S5 and S6). The Photosynthesis–antenna protein pathway (amr00196) showed the strongest enrichment, with 17 of 18 genes differentially expressed and a 7-fold enrichment (adjusted *p* = 1.86 × 10^−12^). The Photosynthesis pathway (amr00195) was also significantly enriched, with 38 of 86 genes upregulated (adjusted *p* = 1.04 × 10^−10^). These results are summarized in Figure 4A,B. Among downregulated genes, enrichment was observed in pathways associated with fatty acid degradation, cationic antimicrobial peptide resistance, and nitrotoluene degradation.

### 2.8. Co-Expression Network Analysis

**Figure 5:**
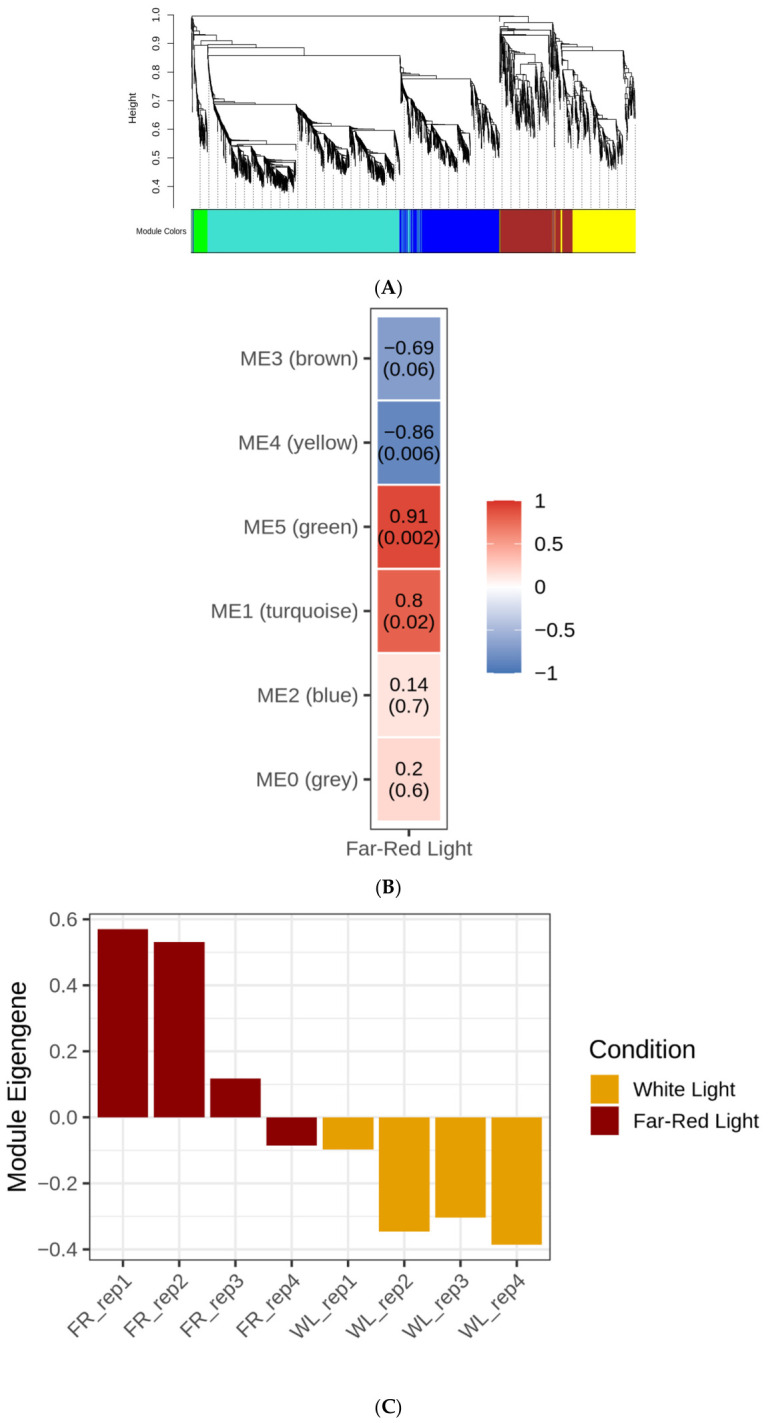
(**A**) Gene dendrogram and module assignments from weighted gene co-expression network analysis of RNA-seq expression data from *A. marina* grown under HL-WL and LL-FR conditions. (**B**) Module–trait relationships between WGCNA modules and LL-FR conditions in *A. marina*. Values shown are Pearson correlation coefficients with corresponding *p* values. (**C**) Eigengene expression values for the turquoise WGCNA module across RNA-seq samples from *A. marina* grown under HL-WL and LL-FR conditions.

Weighted gene co-expression network analysis identified five gene modules based on correlated expression patterns (Figure 5A; Supplementary Figure S7). The turquoise module, containing 1352 genes, included all phycobiliprotein genes from the KEGG amr00196 pathway as well as all additional light-harvesting genes listed in Table 2. This module showed a significant positive correlation with LL-FR conditions (r = 0.802, *p* = 0.017) (Figure 5B). The eigengene expression pattern of this module across samples is shown in Figure 5C. Other modules also displayed strong associations with the experimental conditions, with the green module showing the strongest positive correlation with LL-FR (r = 0.91, *p* = 0.002) and the yellow module showing the strongest negative correlation (r = −0.86, *p* = 0.006). Because the scale-free topology fit of the WGCNA network (R^2^ = 0.66) is below the commonly recommended threshold of 0.80, the module assignments should be interpreted cautiously.

### 2.9. Protein–Protein Interaction Network

Potential functional relationships among differentially expressed genes were explored using the STRING protein–protein interaction database. The resulting interaction network contained 1115 nodes and 4763 edges, significantly exceeding the number expected by chance (PPI enrichment *p* < 1.0 × 10^−16^) (Supplementary Figure S8). Gene ontology enrichment analysis of the network identified biological processes related to photosynthesis, the electron transport chain, photosystem organization, and thylakoid components as significantly overrepresented. However, only 639 of the 1810 differentially expressed genes were mapped to STRING protein identifiers. The remaining 1171 genes, including many plasmid-encoded phycobiliprotein genes, were not in the database. As a result, the interaction network largely reflects proteins encoded by genes on the chromosome, whereas those encoded by plasmids are poorly represented. Together, the combined analyses of differential expression, pathway enrichment, co-expression and protein–protein interactions support a coordinated LL-FR versus *HL-WL* transcriptional response in *Acaryochloris marina*, marked by concerted upregulation of both photosystem genes and phycobiliprotein and chlorophyll-binding antenna systems, with the response of the latter being much greater in magnitude.

## 3. Discussion

### 3.1. Antenna Remodeling Is the Strongest Transcriptional Feature of the LL-FR/HL-WL Contrast

The present analysis indicates that, in the contrast between low-irradiance far-red light (LL-FR) and high-irradiance white light (HL-WL), *Acaryochloris marina* MBIC11017 simultaneously upregulates core photosystem genes and, more strongly, light-harvesting antenna genes. Using GFF-verified locus tags, all eleven PS-I core loci and a majority of PS-II core loci showed significantly increased expression under LL-FR, while the magnitude of induction was substantially greater for antenna-related genes (phycobiliprotein operon, chromosomally encoded Pcb/CBP candidates, and chlorophyll biosynthesis genes; mean antenna log_2_FC = +3.51 versus PS-I +1.96 and PS-II +1.10). Because LL-FR couples a far-red spectral shift with a 15–25-fold reduction in photon flux, the elevated photosystem and antenna transcript levels are most parsimoniously interpreted as a low-irradiance photoacclimation response—expansion of light-harvesting and biogenesis capacity under photon limitation—with a potential CA5-mediated component (via the AM1_5894 bacteriophytochrome) superimposed on phycobiliprotein gene regulation [18,19,20]. This interpretation is consistent with matched-irradiance physiological data showing that, at equal photon flux, far-red illumination decreases rather than increases phycobilisome content [20,21].

This interpretation is consistent with the distinctive photophysiology of *A. marina*, which uses chlorophyll *d* as its dominant pigment and already extends absorption into the far-red region [22,23]. Within the limits of this dataset, the LL-FR/HL-WL response appears less consistent with replacement of core reaction-center components than with increased effective light capture and biogenesis capacity through antenna-associated adjustments.

### 3.2. Strong Induction of Phycobiliprotein Genes Supports Integration of Reacquired Antenna Functions

The clearest transcriptional response in this study was the strong induction of phycobiliprotein genes. Seventeen of the eighteen genes in the KEGG photosynthesis–antenna protein pathway were significantly upregulated under LL-FR, and several showed greater than 20-fold increases in expression. Most of these genes are located on the pREB3 plasmid, consistent with previous work showing that phycobiliprotein genes in *A. marina* MBIC11017 were reacquired by horizontal gene transfer [16,18,19]. Their strong expression under LL-FR therefore supports the idea that these reacquired genes are not peripheral genomic additions, but are functionally engaged during the LL-FR/HL-WL response.

The induction of the chromosomally encoded apcB gene merits separate consideration in light of the unusual phycobilisome (PBS) architecture of *A. marina*. Unlike most cyanobacteria, *A. marina* lacks the core-membrane linker apcE and does not assemble a classical tricylindrical allophycocyanin core; its PBS consist of rod-shaped phycocyanin assemblies in which allophycocyanin subunits constitute only a minor population at the proximal (membrane-facing) end of the rod [11,12,13]. apcB upregulation in this organism is therefore unlikely to reflect remodeling of a canonical APC core. Instead, the most parsimonious interpretation is that apcB participates in the broader low-light-driven induction of phycobiliprotein synthesis observed across both plasmid-borne (cpcA, cpcB, cpcC, cpcD, cpcG, cpcE, cpcF) and chromosomal phycobiliprotein loci, perhaps contributing to the small allophycocyanin population at the rod base or to phycocyanin–rod assembly fidelity. Direct biochemical confirmation of apcB protein abundance, localization, and stoichiometry within rod-only PBS will require complementary proteomic and structural studies. We therefore do not interpret apcB induction as evidence of a canonical core; rather, the result indicates that both plasmid-acquired and ancestral chromosomal phycobiliprotein components are jointly mobilized under the LL-FR condition, consistent with regulatory integration of the horizontally acquired phycocyanin operon into the host light-harvesting program [18,20].

### 3.3. Multiple Antenna Systems Respond Under LL-FR

In addition to phycobiliprotein genes, several strongly induced loci were consistent with previously described chlorophyll-binding Pcb/CBP antenna proteins. These genes are of particular interest because they suggest that LL-FR conditions are associated with increased expression of both water-soluble and membrane-associated antenna systems. Upregulation of *chlL* and *chlN* further supports the possibility of increased pigment-related investment under these conditions, while the strong induction of *psbU* may reflect accompanying changes in PS-II stabilization or assembly.

Taken together, these findings support a model in which *A. marina* responds to the LL-FR versus HL-WL contrast through broad antenna remodeling rather than through a narrowly targeted response in a single light-harvesting subsystem. At the transcript level, the response appears to involve enhancement of both phycobiliprotein-based and chlorophyll-binding antenna capacity.

### 3.4. Relationship to Canonical FaRLiP Responses in Other Cyanobacteria

The transcriptional pattern observed in the LL-FR versus HL-WL contrast differs from the canonical far-red light photoacclimation (FaRLiP) program described in other cyanobacteria. In FaRLiP-capable organisms, far-red growth is associated with replacement of standard photosystem subunits by specialized far-red isoforms encoded within the FaRLiP gene cluster [24,25]. In contrast, the present dataset does not point to a transcript-level response dominated by photosystem core replacement. Instead, it indicates broad increases in photosystem gene expression, accompanied by particularly strong induction of antenna-associated genes. A structured comparison of key features is provided in Table 4.

The comparison should therefore be interpreted cautiously: because irradiance and wavelength are confounded, these data do not define a distinct far-red acclimation mode for *A. marina*. Rather, they show that, in this dataset, the LL-FR/HL-WL transcriptomic response is characterized by increased light-harvesting and photosystem transcript abundance rather than FaRLiP-like replacement of core photosystem subunits.

### 3.5. Network Analyses Provide Supportive but Limited Evidence

The network-based analyses were broadly consistent with the differential expression results, but they should be interpreted cautiously. WGCNA grouped phycobiliprotein genes and additional antenna-associated genes into a module positively associated with LL-FR, supporting the view that these genes share similar expression dynamics across the sampled conditions. However, the limited sample size and the suboptimal scale-free topology fit reduce confidence in the stability of these module assignments. Accordingly, the co-expression results are best regarded as supportive rather than definitive.

STRING analysis likewise identified a significantly enriched interaction network associated with photosynthesis-related functions, including electron transport and thylakoid organization. However, many plasmid-encoded genes, including numerous phycobiliprotein-associated loci, were not represented in the database. The interaction network, therefore, captures only part of the transcriptional response and is weighted toward chromosomally encoded functions. Together, these analyses support the broader interpretation of coordinated transcript-level adjustment, but, by themselves, they do not establish regulatory integration or direct functional interactions among all components.

### 3.6. Limitations and Future Directions

Several limitations require explicit acknowledgment. First, the underlying experiment compared low-irradiance far-red light (1.5–2 µmol photons m^−2^ s^−1^, 710-nm peak) with high-irradiance white light (30–35 µmol photons m^−2^ s^−1^), differing by a factor of approximately 15–25 in photon flux in addition to spectral composition; the effects of light quality and irradiance therefore cannot be separated within this dataset [18]. This limitation is consequential for biological interpretation. Recent matched-irradiance experiments by Oliver et al. [20], conducted at a common photon flux of 20 µmol m^−2^ s^−1^ under fluorescent white versus 740-nm far-red illumination, reported that white-light-grown *A. marina* MBIC11017 contains a higher PSI/PSII ratio and greater phycobilisome content than its far-red-grown counterpart, which has a smaller membrane-embedded antenna pool. Wang et al. [21] similarly reported that, at equal intensity, far-red illumination decreases the PBP/Chl d ratio. Both observations are difficult to reconcile with a wavelength-driven interpretation of the present transcriptional pattern, in which antenna and photosystem genes are upregulated under LL-FR. The most parsimonious explanation is that the bulk of the transcriptional induction reported here reflects a low-light photoacclimation response, that is, an increase in light-harvesting and reaction-center biogenesis under photon limitation, onto which a smaller wavelength-specific component, mediated by the characterized bacteriophytochrome AM1_5894 (the candidate CA5 photoreceptor) [18,19,20], may be superimposed. Second, transcript abundance does not directly demonstrate protein accumulation, antenna stoichiometry, pigment composition, or photosystem assembly state, all of which may diverge from transcript levels under light-shift conditions. Third, network-level analyses are constrained by the small number of biological replicates (*n* = 4 per condition) and by incomplete representation of plasmid-encoded loci in current STRING and KEGG annotations. Fourth, the present manuscript does not aim to redefine type-5 chromatic acclimation (CA5), which is an established mechanism in *A. marina* [16,18,20]; rather, its contribution lies in describing the genome-wide, systems-level architecture of the LL-FR/HL-WL transcriptional response across plasmid-encoded acquired antenna genes, chromosomal Pcb/CBP loci, chlorophyll biosynthesis, and GFF-verified photosystem subunits, while providing a modular co-expression framework for future hypothesis-driven experiments.

Future work should test *A. marina* under disentangled light-quality and irradiance conditions and combine transcriptomic analysis with proteomic, pigment, and physiological measurements. Such experiments will be needed to determine whether the transcriptional patterns observed here correspond to quantitative remodeling of antenna composition, altered photosystem organization, or both.

## 4. Materials and Methods

### 4.1. Data Source and Experimental Design

Paired-end RNA-seq data were obtained from the NCBI Sequence Read Archive under BioProject PRJNA1130970 [18]. The dataset comprised *Acaryochloris marina* MBIC11017 cultures grown under low-irradiance far-red light (LL-FR; 1.5–2 µmol photons m^−2^ s^−1^, LED with 710-nm emission peak) and high-irradiance white light (HL-WL; 30–35 µmol photons m^−2^ s^−1^, cool-white fluorescent), with a 12-h light/dark cycle at 30 °C and four biological replicates per condition (eight libraries in total) [18]. The two conditions therefore differ both in spectral quality and in irradiance (by approximately 15–25), and the original study emphasized that this design limits the separation of irradiance- and spectrum-dependent effects [18]. As this confounding cannot be resolved post hoc, throughout this study, we interpret transcriptional differences as responses to the combined LL-FR versus HL-WL treatment rather than to far-red wavelength alone.

### 4.2. Read Alignment and Quantification

Raw sequencing reads were downloaded from the NCBI SRA using SRA Toolkit v2.11.3 (prefetch and fasterq-dump). Read quality was assessed using FastQC v0.11.5, and no libraries showed quality issues requiring exclusion. Reads were aligned to the *A. marina* MBIC11017 reference genome (GCA_000018105.1) using STAR v2.7.10b [26]. Quality-control summaries were compiled with MultiQC [27]. The resulting BAM files were sorted and indexed with SAMtools v1.6 [28]. Gene-level read counts were generated using featureCounts v2.1.1 [29].

### 4.3. Differential Expression Analysis

Differential expression analysis was performed in R v4.3.3 using DESeq2 v1.42.1 [30,31]. Genes with fewer than 10 reads across fewer than 2 samples were excluded prior to analysis, leaving 8439 genes for statistical testing. Log2 fold changes were shrunk using apeglm v1.24.0 [32]. *p*-values were adjusted for multiple testing using the Benjamini–Hochberg false discovery rate method [33]. Genes with adjusted *p* < 0.05 were considered differentially expressed. Positive log2 fold change values indicate higher expression under LL-FR relative to HL-WL.

### 4.4. Gene Set Analysis

To assess coordinated transcriptional responses, gene set analysis was performed for photosystem I (PS-I; 11 gene loci encoding 10 subunit types), photosystem II (PS-II; 20 gene loci encoding 15 subunit types), and selected light-harvesting antenna gene groups. For each gene set, log2 fold change values were evaluated using complementary statistical approaches, including a one-sample *t*-test against zero, a Wilcoxon-based test, and permutation testing (100,000 iterations), to determine whether the overall expression response of the set differed from the null expectation.

### 4.5. KEGG Pathway Enrichment

KEGG pathway-to-gene mappings for *A. marina* (organism code: amr) were obtained from the KEGG database [34] using the KEGGREST R package (v1.42.0). Enrichment analysis was performed using Fisher’s exact test, with the set of genes retained for differential expression analysis used as the background universe. Resulting *p*-values were adjusted using the Benjamini–Hochberg method, and pathways with adjusted *p* < 0.05 were considered significantly enriched.

### 4.6. Weighted Gene Co-Expression Network Analysis

Weighted gene co-expression network analysis (WGCNA) was performed using variance-stabilized expression values for the 3000 most variable genes [35]. A signed co-expression network was constructed using a soft-threshold power of 20. The resulting scale-free topology fit was R^2^ = 0.66, which is below the commonly recommended threshold of 0.80 and likely reflects, at least in part, the limited sample size. Gene modules were identified using dynamic tree cutting with a minimum module size of 30 and a merge cut height of 0.25. Because only eight samples were available, WGCNA results were interpreted as exploratory and used primarily to identify broad patterns of coordinated expression.

### 4.7. Protein–Protein Interaction Network Analysis

Differentially expressed genes were analyzed using the STRING database v12.0 [36], with *A. marina* MBIC11017 (taxid: 329726) specified as the reference organism. STRING integrates known and predicted functional associations. The submitted differentially expressed gene set, together with first-shell interactors, produced a network containing 1115 nodes and 4763 edges at medium confidence (combined score ≥ 0.400). The STRING protein–protein interaction enrichment *p*-value was used to assess whether the observed network contained more interactions than expected for a random set of proteins of similar size.

## 5. Conclusions

Comparison of high-irradiance white light (HL-WL; 30–35 µmol photons m^−2^ s^−1^) and low-irradiance far-red light (LL-FR; 1.5–2 µmol photons m^−2^ s^−1^) shows that *Acaryochloris marina* MBIC11017 undergoes extensive coordinated transcriptional remodeling. Genes encoding the cores of both photosystem I (11/11 loci) and photosystem II (12/20 loci), essentially the entire KEGG photosynthesis–antenna protein pathway (amr00196; 17/18 genes), and multiple chromosomal loci consistent with chlorophyll-binding Pcb/CBP antenna proteins are upregulated under LL-FR, with antenna-associated genes showing the strongest induction. Co-expression network analysis assigns these genes to a single module strongly correlated with LL-FR, and STRING analysis supports a significantly interconnected interaction network. Because light quality and irradiance cannot be separated in the source experiment, and because matched-irradiance physiological data indicate that far-red light does not enhance phycobilisome content, we interpret the bulk of the transcriptional response as a low-irradiance photoacclimation, with a possible wavelength-specific component layered onto phycobiliprotein regulation. The integrative analysis nonetheless provides a genome-wide, systems-level map of how plasmid-borne reacquired phycobiliprotein genes, ancestral chromosomal antenna components, chlorophyll biosynthesis, and the photosystem cores are co-mobilized under a combined low-light far-red shift, providing a co-expression framework for future experiments designed to disentangle wavelength- and intensity-driven regulation.

## Figures and Tables

**Table 1:** Summary of RNA-seq library statistics for *Acaryochloris marina* MBIC11017 samples grown under high-irradiance white light (HL-WL) and low-irradiance far-red light (LL-FR) conditions.

Sample	Condition	Total Frags	Assigned	Assign%	Size Fac.
WL_rep1	HL-WL	24,147,398	11,006,566	45.6	1.106
WL_rep2	HL-WL	26,066,800	10,531,587	40.4	1.319
WL_rep3	HL-WL	25,616,622	9,203,960	35.9	1.126
WL_rep4	HL-WL	21,912,350	10,675,257	48.7	1.381
FR_rep1	LL-FR	22,500,060	7,639,137	34.0	0.553
FR_rep2	LL-FR	26,549,146	9,872,029	37.2	0.738
FR_rep3	LL-FR	22,809,108	10,173,428	44.6	1.019
FR_rep4	LL-FR	21,037,344	8,968,970	42.6	1.041
Total		190,638,828	78,070,934	41.0	

**Table 2:** Light-harvesting and pigment-associated genes significantly upregulated under LL-FR relative to HL-WL in *Acaryochloris marina* MBIC11017. Genes are grouped by functional category and sorted by fold change within each group.

Gene ID	Gene	Function/Category	Fold Change	log_2_FC	Adjusted *p* Value
**Phycobilisome proteins–KEGG amr00196 pathway (17 genes)**					
AM1_C0098	cpcB	Phycocyanin β subunit	27.5	+4.78	1.39 × 10^−21^
AM1_C0192	cpcB	Phycocyanin β subunit	25.1	+4.65	2.10 × 10^−12^
AM1_C0191	cpcA	Phycocyanin α subunit	14.3	+3.84	3.81 × 10^−10^
AM1_C0096	cpcA	Phycocyanin α subunit	12.0	+3.58	7.44 × 10^−08^
AM1_C0100	cpcB	Phycocyanin β subunit	11.1	+3.48	8.47 × 10^−11^
AM1_C0099	cpcA	Phycocyanin α subunit	10.9	+3.44	5.14 × 10^−09^
AM1_C0212	cpcB	Phycocyanin β subunit	10.3	+3.37	1.91 × 10^−12^
AM1_C0203	cpcG	PC rod–rod linker	8.8	+3.14	1.79 × 10^−09^
AM1_C0216	cpcD	PC rod cap linker	8.3	+3.06	2.54 × 10^−12^
AM1_C0094	cpcC	PC rod–core linker	6.6	+2.72	2.34 × 10^−07^
AM1_C0102	cpcG	PC rod–rod linker	5.7	+2.52	2.32 × 10^−06^
AM1_C0213	cpcA	Phycocyanin α subunit	5.2	+2.37	3.20 × 10^−07^
AM1_C0215	cpcC	PC rod–core linker	4.8	+2.26	1.36 × 10^−06^
AM1_C0093	cpcD	PC rod cap linker	4.7	+2.24	1.56 × 10^−07^
AM1_2376	apcB	Allophycocyanin β subunit	2.5	+1.29	5.14 × 10^−03^
AM1_C0118	cpcE	Phycocyanobilin lyase	2.1	+1.05	2.81 × 10^−05^
AM1_C0272	cpcF	Bilin attachment protein	2.0	+1.03	9.04 × 10^−07^
**Putative Pcb/*CBP* membrane antenna proteins**					
AM1_5044	—	Putative Pcb/CBP antenna protein (chromosome)	35.3	+5.14	5.42 × 10^−15^
AM1_3688	—	Putative Pcb/CBP antenna protein (chromosome)	13.9	+3.79	1.97 × 10^−13^
AM1_3687	—	Putative Pcb/CBP antenna protein (chromosome)	13.7	+3.78	1.52 × 10^−15^
AM1_3689	—	Putative Pcb/CBP antenna protein (chromosome)	12.2	+3.61	3.85 × 10^−14^
AM1_3686	—	Putative Pcb/CBP antenna protein (chromosome)	11.1	+3.47	1.22 × 10^−19^
**Chlorophyll biosynthesis–DPOR subunits**					
AM1_1445	*chlL*	DPOR subunit, Chl biosynthesis (chromosome)	12.3	+3.63	1.77 × 10^−35^
AM1_1444	*chlN*	DPOR subunit, Chl biosynthesis (chromosome)	8.5	+3.09	1.71 × 10^−26^
**Uncharacterized pREB3 genes**					
AM1_C0097	—	Uncharacterized (pREB3)	10.0	+3.32	7.23 × 10^−08^
AM1_C0095	—	Uncharacterized (pREB3)	9.9	+3.30	6.07 × 10^−16^
AM1_C0214	—	Uncharacterized (pREB3)	8.4	+3.08	4.39 × 10^−11^
AM1_C0189	—	Uncharacterized (pREB3)	4.3	+2.09	1.48 × 10^−15^
**PS-II associated ^a^**					
AM1_5046	*psbU*	PS-II extrinsic subunit (amr00195)	31.8	+4.99	2.85 × 10^−26^

Note: Fold change represents expression in LL-FR relative to HL-WL. Positive log_2_FC values indicate higher expression under LL-FR relative to HL-WL. Adjusted *p* values from Benjamini–Hochberg correction. One gene in the phycobilisome group is chromosomally encoded: apcB (AM1_2376). All other phycobilisome genes and all pREB3 genes are plasmid-encoded. ^a^ psbU (AM1_5046) encodes a PS-II extrinsic subunit (KEGG amr00195) that stabilizes the oxygen-evolving complex. Its marked upregulation (31.8-fold) is consistent with increased PS-II assembly under the LL-FR condition. It is listed separately to distinguish it from the core antenna components.

**Table 3:** KEGG pathway enrichment analysis of differentially expressed genes in *Acaryochloris marina* under low-irradiance far-red light (LL-FR) relative to high-irradiance white light (HL-WL). Pathways enriched among upregulated and downregulated genes were identified using KEGG pathway analysis. Enrichment values represent fold enrichment relative to the background gene set. Arrows indicate enrichment among genes upregulated (↑) or downregulated (↓) under LL-FR conditions.

Pathway	Description	Total Genes in Pathway	DE Genes	Fold Enrichment	Adjusted *p* Value
amr00196	Photosynthesis–antenna proteins ↑	18	17/18	7.0	1.86 × 10^−12^
amr00195	Photosynthesis ↑	86	38/86	3.3	1.04 × 10^−10^
amr01100	Metabolic pathways ↑	796	147/796	1.4	4.84 × 10^−04^
amr00071	Fatty acid degradation ↓	10	5/10	6.2	2.33 × 10^−02^
amr01503	CAMP resistance ↓	15	6/15	5.0	2.33 × 10^−02^
amr00633	Nitrotoluene degradation ↓	4	3/4	9.4	4.34 × 10^−02^

↑ = enriched among upregulated genes; ↓ = enriched among downregulated genes.

**Table 4:** Comparison of the *Acaryochloris marina* LL-FR/HL-WL transcriptional response with canonical FaRLiP cyanobacteria.

Feature	*A. marina* (LL-FR vs. HL-WL; This Study)	FaRLiP Cyanobacteria [24,25]
Primary far-red pigment	Chlorophyll *d* (constitutive)	Chlorophyll *f* (induced)
Core photosystem gene response	Transcript levels significantly upregulated under LL-FR relative to HL-WL (PS-I 3.9×, 11/11 gene loci; PS-II 2.1×, 12/20 gene loci)	Replacement of standard photosystem subunits with far-red-specific isoforms
Antenna response	Transcript upregulation of phycobiliproteins and Pcb/CBP antenna proteins	Remodeling of antenna complexes, including far-red–specific allophycocyanin variants
KEGG antenna pathway (amr00196)	17 of 18 phycobiliprotein genes are differentially expressed	Not characterized
Protein–Protein Interaction network	1115 nodes, PPI enrichment *p* < 1.0 × 10^−16^	Not characterized
Overall interpretation	Predominantly low-irradiance photoacclimation in the LL-FR/HL-WL contrast, with possible CA5 contribution	Photosystem subunit switching via the FaRLiP gene cluster

## Data Availability

RNA-seq data: PRJNA1130970 [18]. Reference genome: GCA_000018105.1. STRING v12.0, taxid 329726.

## Supplementary Materials

The following supporting information can be downloaded at: https://www.mdpi.com/article/10.3390/plants15111605/s1, Figure S1: Pearson sample correlation matrix; Figure S2: Top 50 DE genes heatmap (Z-scored VST counts); Figure S3: MA plot; Figure S4: DESeq2 dispersion estimates; Figure S5: KEGG enrichment among all DE genes; Figure S6: KEGG enrichment among downregulated genes; Figure S7: WGCNA soft threshold selection (power 20, R^2^ = 0.66); Figure S8: STRING protein–protein interaction (PPI) network of differentially expressed genes in *Acaryochloris marina*.

## Abbreviations

Chl	chlorophyll
PS-I	photosystem I
PS-II	photosystem II
Pcb	prochlorophyte chlorophyll-binding protein
CBP	chlorophyll-binding protein
CA5	chromatic acclimation type 5
LL-FR	low-irradiance far-red light
HL-WL	high-irradiance white light
DE	differentially expressed
KEGG	Kyoto Encyclopedia of Genes and Genomes
WGCNA	weighted gene co-expression network analysis
PPI	protein–protein interaction
HGT	horizontal gene transfer
FaRLiP	far-red light photoacclimation
DPOR	dark-operative protochlorophyllide oxidoreductase
VST	variance-stabilizing transformation
