# Supplementary material for: Transcriptomic Remodeling of Light Harvesting and Photosystem Genes in Acaryochloris marina Under a Low-Irradiance Far-Red Versus High-Irradiance White Light
Source: Plants (Basel). 2026 May 23;15(11):1605. doi: 10.3390/plants15111605 (PMC13259208; doi:10.3390/plants15111605)
Supplement: Supplementary file 1 [file plants-15-01605-s001.zip › plants-4269211-supplementary.pdf]

Supplementary Information

Transcriptomic Remodeling of Light Harvesting and Photosystem Genes in *Acaryochloris marina* Under a Low-Irradiance Far-Red Versus High-Irradiance White Light

Abraham Peele Karlapudi[1], Vuyyuru Kesavi Himabindhu[1] and Divya Kaur[2,*]

[1]Department of Biotechnology, Vignan's Foundation for Science, Technology and Research, Guntur 522213, Andhra Pradesh, India

[2]Department of Chemistry, Brock University, St. Catharines, ON L2S 3A1, Canada

Correspondence author: dmatta@brocku.ca

S1. Pearson sample correlation matrix.

S2. Top 50 DE genes heatmap (Z-scored VST counts).

S3. MA plot.

S4. DESeq2 dispersion estimates.

S5. KEGG enrichment among all DE genes.

S6. KEGG enrichment among downregulated genes.

S7. WGCNA soft threshold selection (power 20, R^2 = 0.66).

S8. STRING protein–protein interaction (PPI) network of differentially expressed genes in *Acaryochloris marina*.

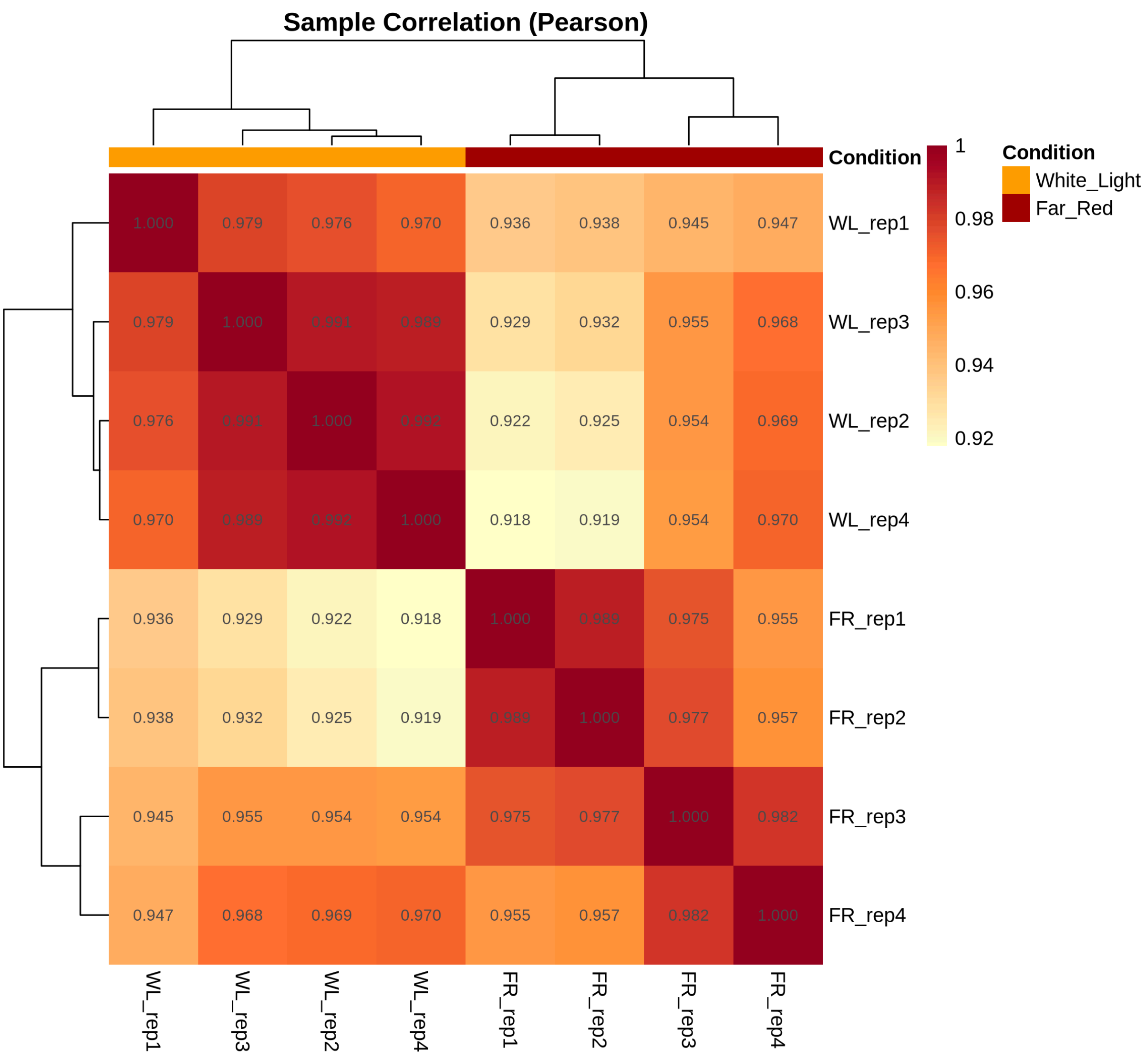

Supplementary Figure S1. Pearson sample correlation matrix. Pairwise Pearson correlation coefficients between RNA-seq samples computed from variance-stabilized transformed (VST) count data. All replicates within each condition show high within-group correlation ($r > 0.99$). WL: white light; FR: far-red light.

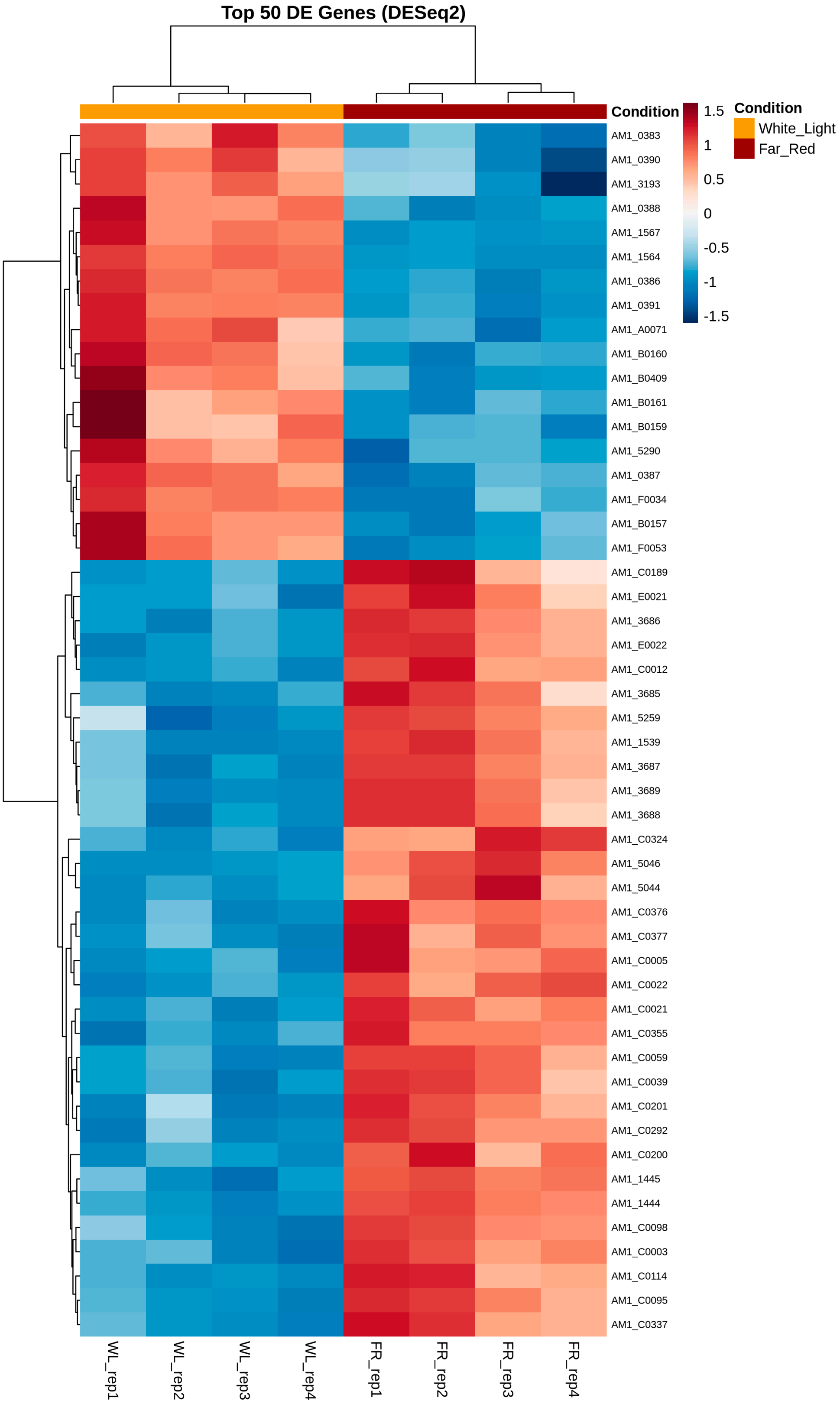

Supplementary Figure S2. Top 50 DE genes heatmap (Z-scored VST counts). Heatmap of the top 50 differentially expressed genes ranked by adjusted P value. Counts are variance-stabilized and Z-score normalized per row. Columns represent individual RNA-seq samples. Clustering reveals clear separation between white light (WL) and far-red light (FR) conditions.

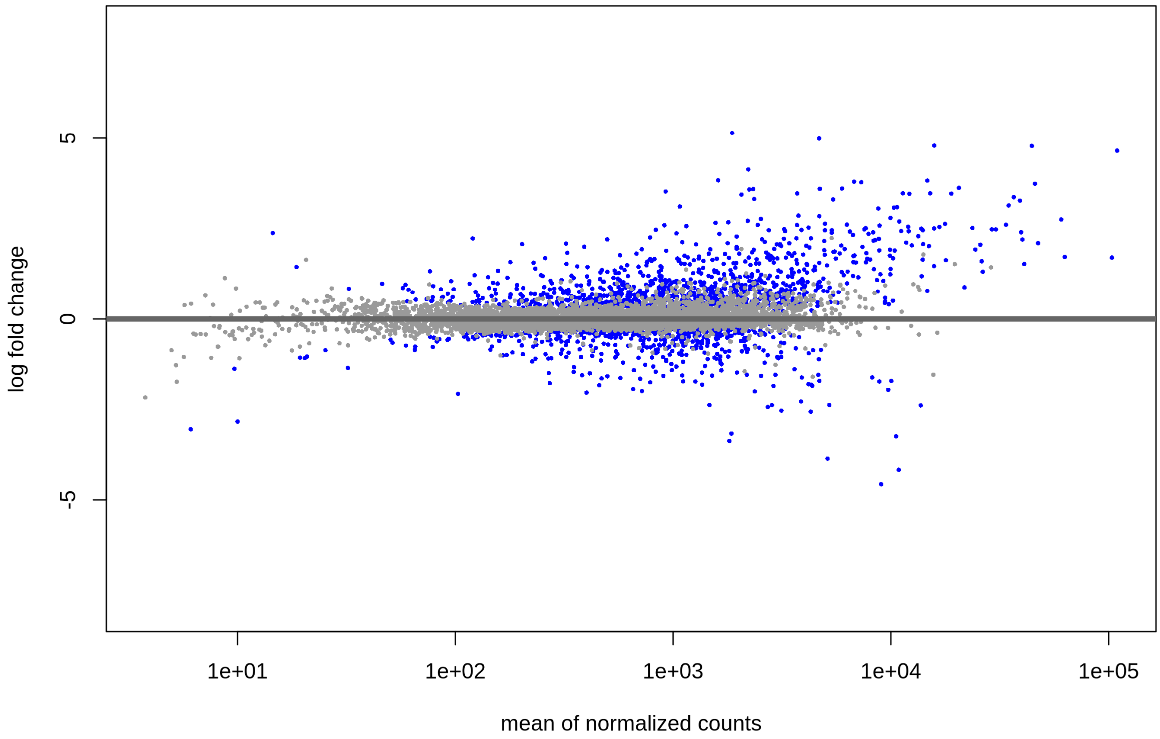

Supplementary Figure S3. MA plot. MA plot showing mean normalized expression (x-axis) versus $\log_2$ fold change (y-axis) for all 8,439 genes tested. Red points indicate genes significantly differentially expressed at adjusted $P < 0.05$.

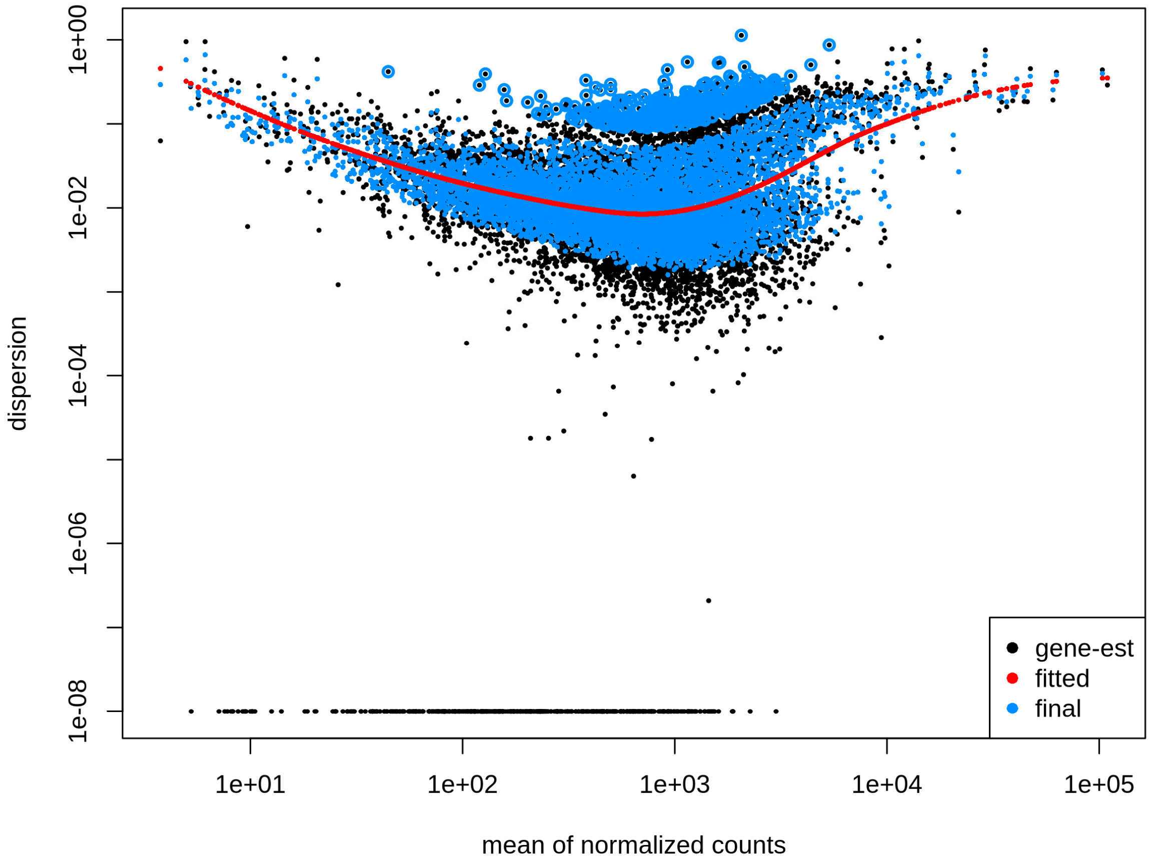

Supplementary Figure S4. DESeq2 dispersion estimates. Dispersion estimates for all genes plotted against mean normalized counts. Gene-wise estimates (black dots), fitted dispersion trend (red curve), and final shrunken estimates (blue dots) are shown. The fitted trend confirms appropriate dispersion modeling by DESeq2.

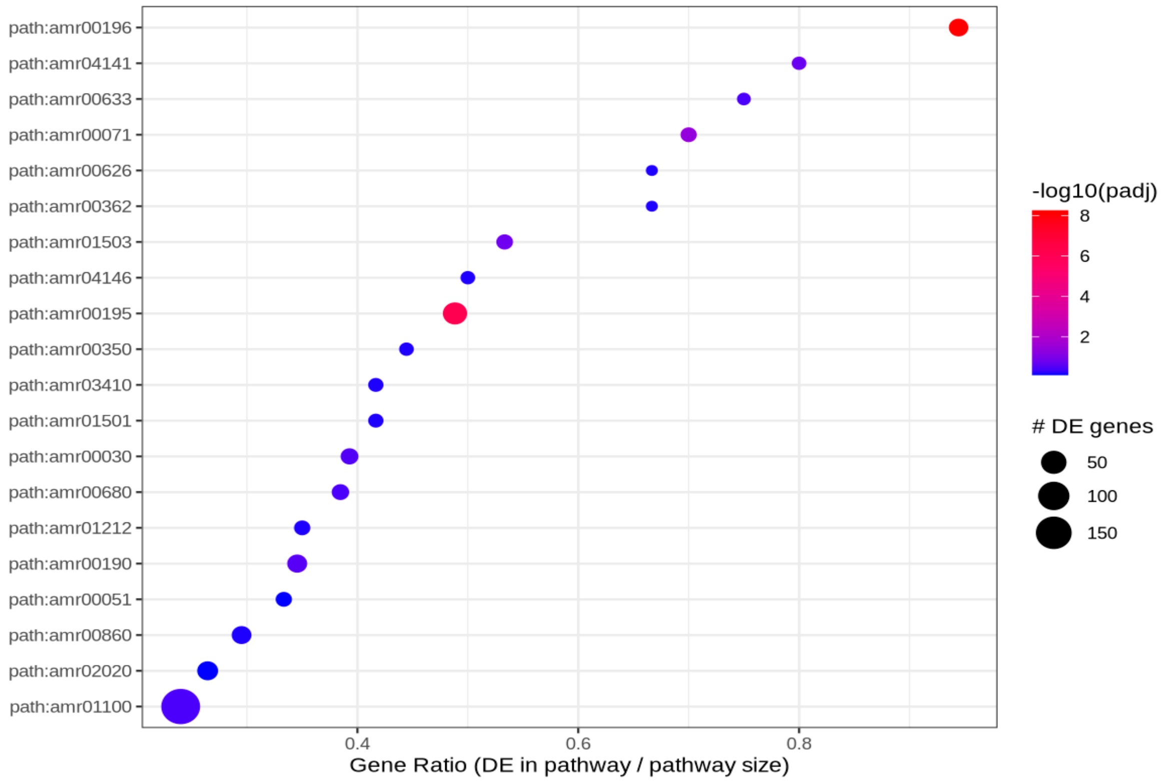

Supplementary Figure S5. KEGG enrichment among all DE genes. Bubble plot showing KEGG pathways significantly enriched (adjusted P < 0.05) among all differentially expressed genes (both upregulated and downregulated) in *Acaryochloris marina* under far-red light conditions. Bubble size represents number of DE genes per pathway; color indicates adjusted P value.

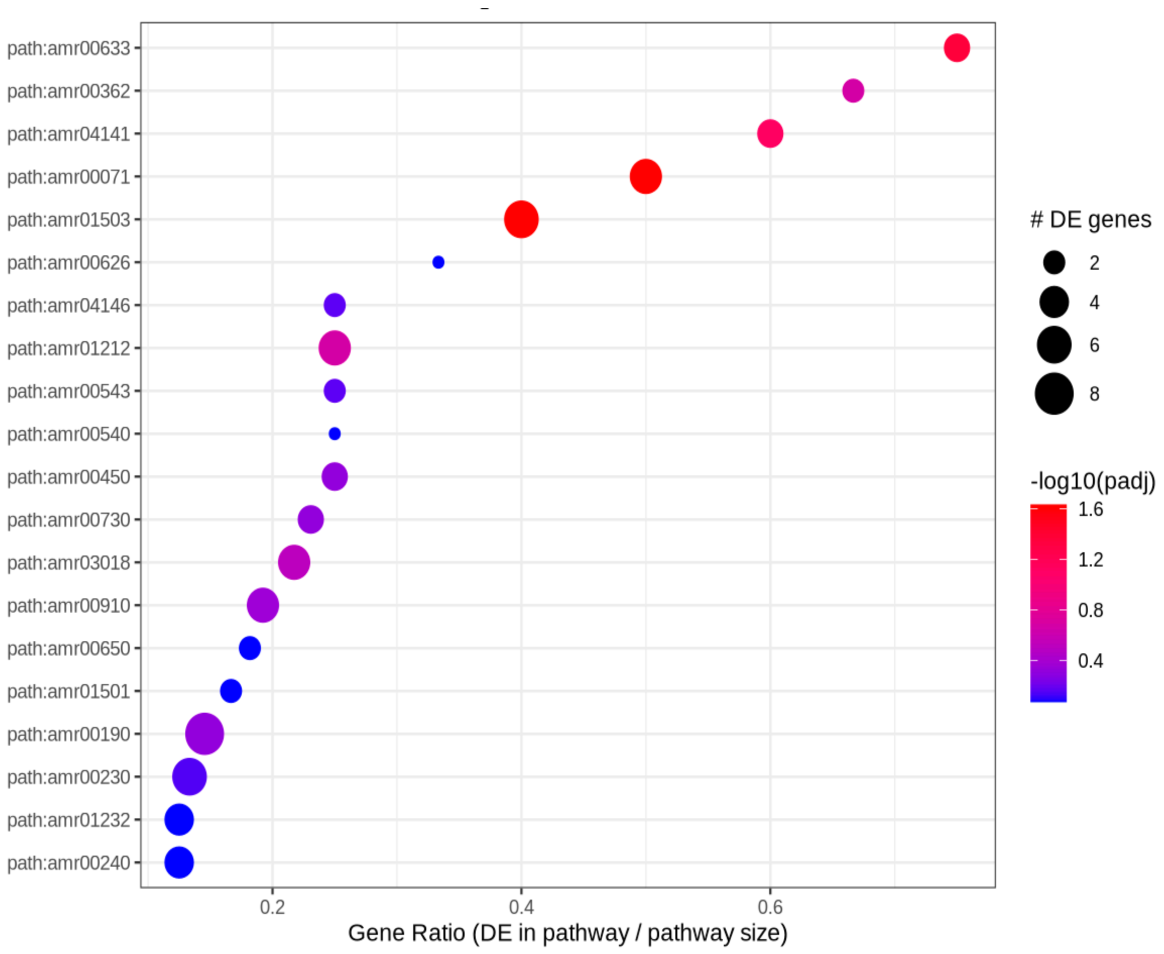

Supplementary Figure S6. KEGG enrichment among downregulated genes. Bubble plot showing KEGG pathways significantly enriched among genes downregulated under far-red light in *Acaryochloris marina*. Enrichment was assessed using hypergeometric testing with Benjamini-Hochberg correction.

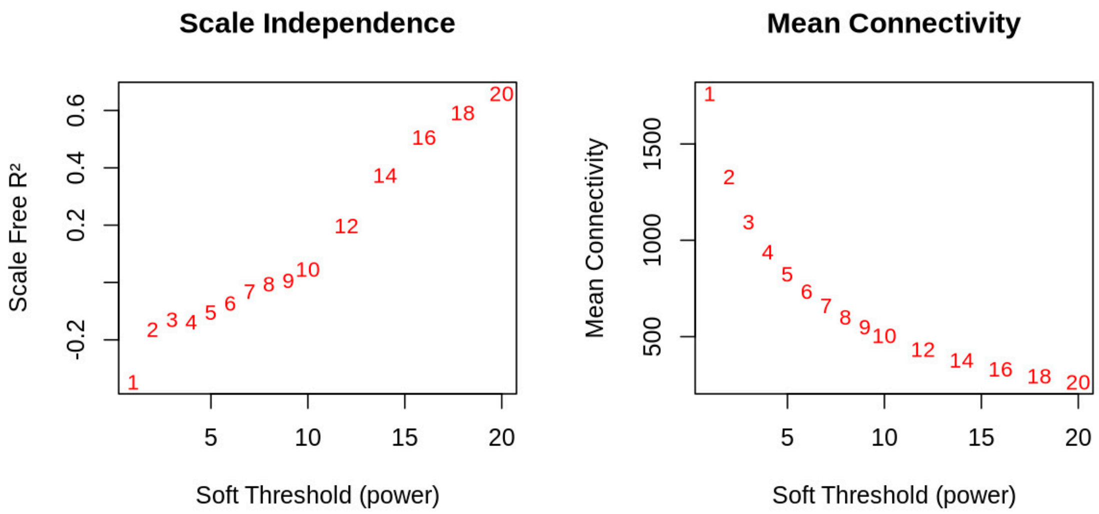

Supplementary Figure S7. WGCNA soft threshold selection (power 20, R^2 = 0.66). Scale-free topology model fit (R^2, left) and mean connectivity (right) as a function of soft-thresholding power. Power 20 (dashed red line) was selected as the lowest power achieving $R^2 \geq 0.66$, satisfying scale-free topology criteria for the 8-sample dataset.

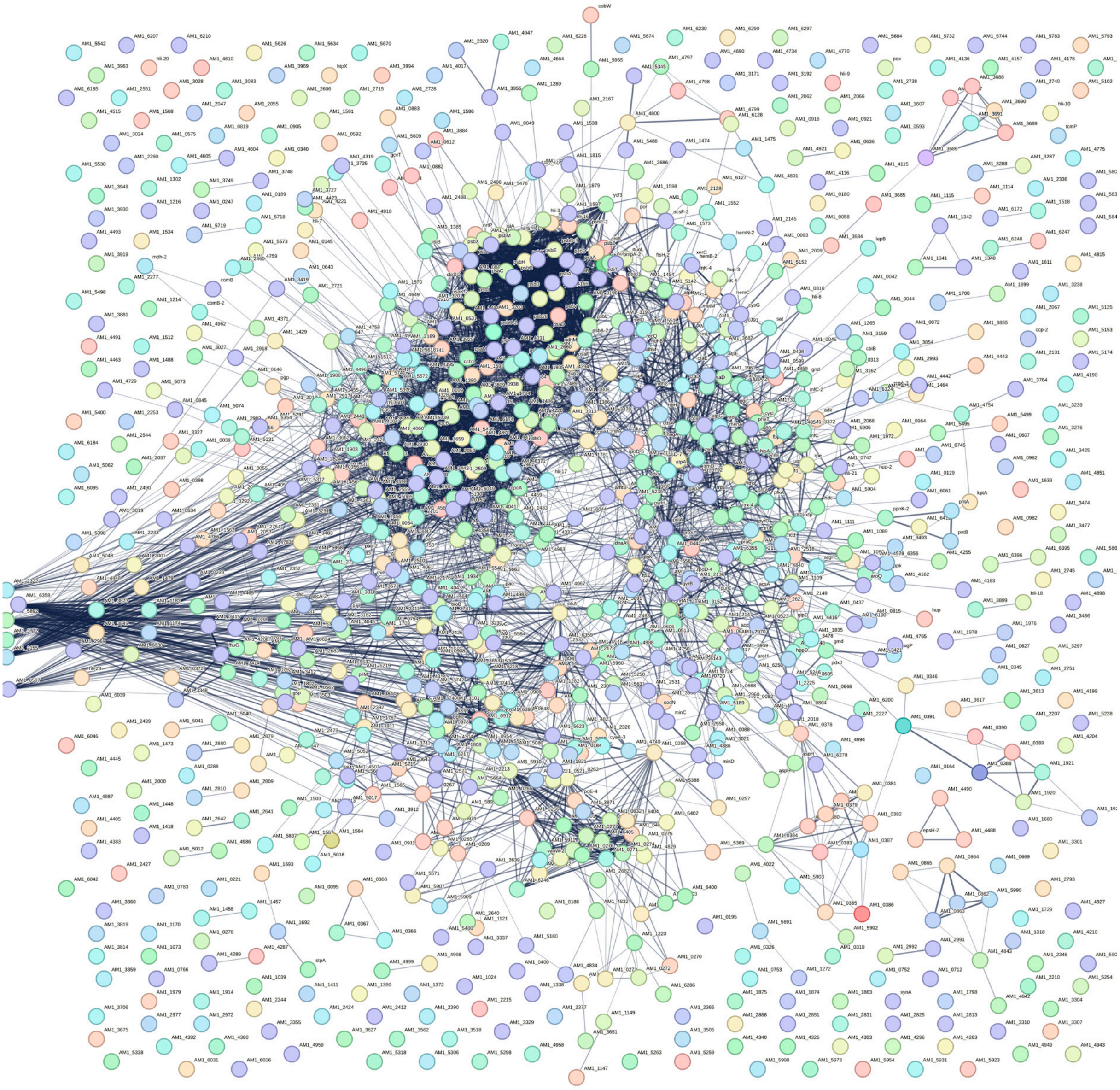

Supplementary Figure S8. STRING protein–protein interaction (PPI) network of differentially expressed genes in *Acaryochloris marina*. Differentially expressed genes identified from RNA-seq analysis were queried against the STRING database (v12.0) to evaluate functional connectivity. The resulting network, including first-shell interactors, contains 1,115 nodes and 4,763 edges (PPI enrichment $P < 1.0 \times 10^{-16}$). Node colors represent STRING functional clusters.
